# Local and Regional Determinants of an Uncommon Functional Group in Freshwater Lakes and Ponds

**DOI:** 10.1371/journal.pone.0131980

**Published:** 2015-06-29

**Authors:** Michael James McCann

**Affiliations:** Department of Ecology and Evolution, Stony Brook University, Stony Brook, New York, United States of America; University of Siena, ITALY

## Abstract

A combination of local and regional factors and stochastic forces is expected to determine the occurrence of species and the structure of communities. However, in most cases, our understanding is incomplete, with large amounts of unexplained variation. Using functional groups rather than individual species may help explain the relationship between community composition and conditions. In this study, I used survey data from freshwater lakes and ponds to understand factors that determine the presence of the floating plant functional group in the northeast United States. Of the 176 water bodies surveyed, 104 (59.1%) did not contain any floating plant species. The occurrence of this functional group was largely determined by local abiotic conditions, which were spatially autocorrelated across the region. A model predicting the presence of the floating plant functional group performed similarly to the best species-specific models. Using a permutation test, I also found that the observed prevalence of floating plants is no different than expected by random assembly from a species pool of its size. These results suggest that the size of the species pool interacts with local conditions in determining the presence of a functional group. Nevertheless, a large amount of unexplained variation remains, attributable to either stochastic species occurrence or incomplete predictive models. The simple permutation approach in this study can be extended to test alternative models of community assembly.

## Introduction

How are ecological communities assembled from the available species pool? What explains the presence or absence of a species at a particular site? Evolutionary processes such as speciation and extinction shape a regional species pool, while processes acting on regional (e.g., dispersal) and local (e.g., abiotic suitability, biotic interactions) scales filter that regional species pool into a smaller set of co-occurring species that form the local community [1]. Generally, ecologists are interested in the relative strength of local and regional processes in structuring communities and whether these processes act deterministically or are stochastic. Typically, both local and regional factors are found to affect community structure [2], but there is variation in the strength of these forces depending on the spatial [3] or temporal [4] scale of the study, community age [5], focal taxa [6], and particular traits of the taxa (e.g., dispersal-related traits [7]). In many cases, our understanding of what controls species occurrence or community assembly is incomplete, with large amounts of variation left unexplained [2].

Another important aspect of community structure is functional group diversity. The diversity of functional groups (i.e., the number of different groups) can also have significant impacts on communities and ecosystems [8]. In fact, the presence or absence of a single functional group can dramatically change community and ecosystem processes. For example, the gain of nitrogen-fixing plants significantly altered ecosystem development after volcanic eruptions in Hawaii [9]. In the Caribbean, the loss of large herbivores contributed to a community shift on reefs dominated by corals to dominance by macroalgae [10]. Although functional groups are widely accepted as important in natural communities, we do not know the relative contributions of local, regional, or biogeographic processes on the occurrence of whole functional groups, especially in freshwater systems.

Although species are categorized into functional groups based on functional similarity, the species may not respond uniformly to the local and regional processes that act as filters on the species pool. Furthermore, the prevalence of a functional group may be influenced by the number of species that are members of the group. Simply through random sampling, a functional group with few species should be less likely to have a representative species in a community than a functional group with a larger number of species. Therefore, processes acting on biogeographic scales that shape the species pool will influence the diversity of functional groups found in a community.

Freshwater lakes and ponds are particularly useful for addressing questions about community structure and assembly because they are well-defined ecosystems that exist in a matrix of unsuitable habitat and have been the focus of a number of studies of community assembly and structure [11]. Previous work that focused on species level diversity has shown that a combination of local and regional processes shape community assembly and composition in freshwater lakes and ponds [12–13], while other work has found local factors more influential than regional factors [14–15]. In many cases, the answer depends on the taxa [6–7] or spatial scale [16] of interest. Furthermore, most studies find that a large portion of the variation in community composition is unexplained [4, 15, 17]. Using functional groups, rather than individual species, may reduce the unexplained variation between community composition and conditions, because species within a functional group may be governed by similar assembly processes. A focus on functional groups will be especially useful if the members of the group are not only functionally similar (i.e., effect traits), but also respond similarly to biotic and abiotic conditions (i.e., response traits) and have similar dispersal abilities [18].

Aquatic plants are typically separated into four functional groups based on their growth form and position in the water column [19–20]: submerged species are rooted in the sediment with the majority of their structures below the water surface; emergent species are rooted in the sediment and the majority of their structures are above the water; floating-attached species, such as lilies, are anchored into the sediment, but their leaves and flowers float on the surface of the water; and free-floating species (hereafter, floating plants) are not anchored into the sediment, and float at or near the water surface. Floating plants take up nutrients directly from the water column, and like floating-attached species, shade the water column below. In small water bodies with high nutrient levels, floating plants can become dominant and cover the entire surface of a water body, and as the superior competitors for light, they can replace submerged species as the dominant primary producers in the water body [21–24]. Water bodies dominated by floating plants have lower dissolved oxygen levels, support a less diverse biota, and have a lower recreational value [25]. The prevalence of the floating plant functional group (i.e., the percentage of water bodies occupied by floating plants) varies regionally. While it has been shown previously that ponds or lakes lacking this functional group are rare [26] (Minneapolis, Minnesota, USA), other studies have found floating plants to be sporadic [27] (Ontario, Canada) or more often absent than not (McCann *personal observations*; Connecticut and Long Island, New York, USA). The cause of this regional variation is unclear.

I used survey data from 176 freshwater lakes and ponds in Connecticut, USA to test the relative importance of local and regional factors in determining the prevalence of the floating plant functional group and its constituent species. I also developed a null model to compare the observed occurrence of this functional group to an expectation based solely on the size of the species pool with individual species prevalence drawn randomly from all aquatic plant species and without regard to competitive interactions or niche similarity. Finally, I compared the prevalence and species richness of floating plants in Connecticut, USA to published records of aquatic plant species in eight other regions of the world to determine if the pattern observed in the northeast USA is typical for floating plant communities around the world.

## Methods

### Connecticut, USA survey data

I acquired data on the presence and absence of aquatic plant species and environmental variables in 176 lakes and ponds in Connecticut, USA from the Connecticut Agricultural Experiment Station (CAES) Invasive Aquatic Plant Program. The CAES collected these data to document the occurrence of native and non-native plants in aquatic ecosystems. These data span a variety of types of freshwater lakes and ponds across the state. Surveys were conducted by CAES between 2005 and 2013 on at least one occasion in each water body. I retrieved all data published on the CAES website (http://www.ct.gov/caes/) by December 1, 2014. For water bodies with more than one survey, I averaged variables across dates and assumed that all plants species observed at least once in a water body were present in that water body. CAES measured a number of local, abiotic variables including conductivity, pH, alkalinity, and total phosphorus at a depth of 0.5 m below the water surface and 0.5 m above the bottom of the deepest portion of the lake. For each parameter, I averaged the samples from both depths to get a single value for each water body. The surface area, the maximum depth, and the latitude and longitude of the water body were also reported by CAES (Table 1).The CAES data also included the species richness of other aquatic plant species (i.e., not floating plants) (Table 1). Unfortunately, this was the only biotic variable that could be incorporated in this analysis due to limitations of the available data.

**Table 1:** Predictor variables used in generalized linear models of species or functional group presence.

Variable	Units	Code	Source
Water body surface area	ha	size	CAES
Shoreline development index		shoreline	CAES *
Depth, maximum	m	depth	CAES
Total phosphorus, average	mg L^-1^	totalP	CAES
pH, average		pH	CAES
Conductivity, average	μS cm^-1^	cond	CAES
Alkalinity, average	mg L^-1^	alk	CAES
Secchi depth	m	secchi	CAES
Richness of non-floating plants		nonFP	CAES
Latitude	decimal degrees	latitude	CAES
Longitude	decimal degrees	longitude	CAES
Numb. water bodies within 1 km		lakes1km	ArcGIS, USGS NHD
Numb. water bodies within 10 km		lakes10km	ArcGIS, USGS NHD
Distance to nearest water body with *L*. *minor*	km	distLM	ArcGIS, CAES
Distance to nearest water body with *S*. *polyrhiza*	km	distSP	ArcGIS, CAES
Distance to nearest water body with *Wolffia* sp.	km	distW	ArcGIS, CAES
Boat launch present?		boatlaunch	ArcGIS, CT DEEP

Abbreviations: Connecticut Agricultural Experiment Station (CAES), US Geological Survey National Hydrography Dataset (USGS NHD), Connecticut Department of Energy and Environmental Protection (CT DEEP), *Lemna minor* (LM), *Spirodela polyrhiza* (SP), and *Wolffia* sp. (W)

*measured in Adobe Acrobat

To calculate shoreline development index, a measure of the complexity of the shoreline, I obtained aerial photographs of each water body from the CAES website and measured the shoreline length (*L*) of each water body using the measurement tools in Adobe Acrobat. The shoreline development index was calculated as $L\;/\;(2\;\sqrt{\pi A})$ where *A* is the surface area reported by CAES. I used ArcGIS to calculate a number of regional-scale, spatial predictors (Table 1). The coordinates of all water bodies were superimposed on GIS data layers of surface hydrology from the US Geological Survey National Hydrography Dataset (USGS NHD) and other data layers from the Connecticut Department of Energy and Environmental Protection (CT DEEP). For each surveyed water body, I measured the number of lakes or ponds within 1 km and within 10 km, as a water body’s degree of isolation may affect the number of propagules transported to the water body. Isolated water bodies may be less likely to receive floating plant propagules from neighboring lakes and ponds. To account for recreational boating as a mechanism of dispersal, I determined whether a boat launch was present in each water body by matching surveyed water bodies with the location of boat launches reported by the CT DEEP (http://www.ct.gov/deep). For each water body, ArcGIS was also used to determine the shortest distance to another water body with each of the three most common floating plant taxa (*Lemna minor*, *Spirodela polyrhiza*, and *Wolffia* spp.). Most lakes and ponds in this survey are not connected to each other via rivers or streams, so hydrological connectivity was not included in this analysis. Data are available in S1 Dataset.

### Statistics

I used generalized linear models to test the effect of local and spatial predictor variables (Table 1) on the presence of the floating plant functional group and the presence of each of the three most common floating plant taxa in this region (*Lemna minor*, *Spirodela polyrhiza*, and *Wolffia* sp.). Records of *Wolffia* spp. were aggregated to the genus level because a majority of observations (12 of 23) only identified the plants to the genus *Wolffia*. The presence or absence of each species and the entire functional group was modeled with a logistic regression (binomial error and logit link functions). Predictor variables were ranged to span from 0 to 1 to ensure that the scale of measurement did not influence the interpretation of model coefficients (R package scales, function rescale). For the presence or absence of each species, I included the distance to the nearest water body with that focal species as a predictor variable in the model. Results of a similar analysis for the species richness of floating plants (rather than presence of each species or the functional group) are reported in S1 File.

For each response variable, I ran all possible combinations of the full model of all predictor variables (R package MuMIn, function dredge). Preliminary analysis found that there was no single best model; therefore, I used a model averaging approach. I did not include interactions between predictors because no strong interaction terms were found in preliminary analysis and interactions would have made the number of models to analyze intractable. Since models with ΔAIC_c_ < 2 are typically considered to have similar statistical support [28], I then determined the weighted model average coefficients from all models with ΔAIC_c_ < 2 (R package MuMIn, function model.avg) [28]. The threshold value to transform the predicted probability of occurrence to a binary presence (1) or absence (0) was determined for each response variable by varying this threshold and finding the value that maximizes sensitivity and specificity of the model [29] (R package PresenceAbsence, function optimal.thresholds).

To assess the performance of the average model, I calculated a confusion matrix to compare the observed presences and absences to the model predictions for each response variable. I also calculated the kappa statistic for each model, which measures whether the agreement of observations and predictions is greater than expected due to chance alone [30]. To visualize the performance of the average model across the region, I mapped the predicted probabilities of occurrence (0–1) and the observed absences and presences (0, 1). Finally, to determine the amount of unexplained variance, I calculated the pseudo-R^2^ value according to [31] for each of the best models (i.e., lowest AIC_c_) (R package MuMIn, function r.squaredLR).

Based on preliminary analyses, one observation was removed from the generalized linear models because of a high Cook’s distance (close to 1), suggesting a large influence on the model results [32]. The predictor variables alkalinity and maximum depth were removed because of their large correlations with conductivity and pH and Secchi depth and water body size, respectively (S1 Fig).

To characterize the environmental similarity of water bodies, I used Principal Component Analysis (PCA) to determine the most important sources of variation in local water body conditions in the data set. Variables were centered and scaled prior to analysis. Scores for the first two principal components were then mapped to qualitatively assess the spatial pattern of water body conditions across the study area.

### Permutation: Species pool size

I used a permutation to test the role of the size of the functional group in the occurrence of floating plants. In particular, I wanted to determine if the number of water bodies without floating plants was different than expected based on a null expectation based only on the size of the species pool of floating plants. For each of the six observed floating plant species in Connecticut, I randomly sampled (with replacement) a frequency of occurrence (i.e., number of water bodies found in) from the observed frequencies of occurrence of all 124 aquatic plant taxa in Connecticut (S2 Fig, S1 Table). I randomly assigned each floating plant species to the 176 water bodies based on its randomly sampled frequency of occurrence. For each water body, I determined whether or not a floating plant species was present, and then I calculated the proportion of the 176 water bodies without floating plants. I repeated this re-sampling method 2000 times and compared the observed percentage of water bodies without floating plants to this null expectation. To test how the size of the species pool affected the number of water bodies expected to be occupied by a particular functional group, I repeated the above permutation for species pools of 1, 12, and 24 floating plant species, representing both larger and smaller species pools. This permutation test assumes random community assembly (i.e., no competitive interactions or environmental filtering) and that the commonness of individual species is randomly sampled from all aquatic plant species.

### Comparison to other regions

To determine if the prevalence of floating plants in Connecticut is different from other regions of the world, I compared the percentages of water bodies without floating plants and the size of the floating plant species pool in Connecticut, USA to other regions. Through a literature search and previous knowledge of available data sets, I found data on the prevalence of aquatic plants, including floating plant species, in eight other regions (Table 2). Three of the data sets only reported the number of water bodies in which each species was found and not the list of species in each water body. Therefore, I could not determine the number of water bodies in which floating plants were completely absent. The remaining five data sets reported the list of species in each water body, so it was possible to determine the number of water bodies without floating plants and the species richness of floating plants in each water body.

**Table 2:** Characteristics of aquatic plant communities in other regions of the world.

Location	Spatial extent of survey (km^2^)	# water bodies surveyed	Total aquatic plant species richness	Floating plant richness	% water bodies without floating plants
France [33]	50	23	83	2	73.91
Finland [34]	550	57	91	9	38.60
Saaremaa Island (Estonia) [35]	2750	40	47	2	
Connecticut, USA *	14000	176	124	6	59.10
Northern Ireland [36]	14000	574	85	3	
Norway [17]	17600	64	47	3	20.31
Denmark [37]	43000	82	103	1	81.71
Great Britain, 1989 [38]	89000	1124	102	7	
Great Britain, 2004 [38]	89000	3447	101	8	
Washington, USA ^¶^	185000	502	212	5	76.49

Data sources are indicated by footnotes. Missing values resulted from studies that did not report the list of species in each water body.

* This study

^¶^ Washington State Department of Ecology

## Results

Overall, six taxa of floating plants were observed in the 176 lakes and ponds surveyed in Connecticut. The three most common taxa, *Lemna minor*, *Spirodela polyrhiza*, and *Wolffia* spp., which were the focus of this analysis, occurred in 56, 31, and 23 water bodies, respectively. Less common species of floating plants were *Azolla* sp., *Eicchornia crassipes*, and *Lemna trisulca* (which often floats slightly below the water surface), all of which occurred in four or fewer water bodies. Of the 176 water bodies, 104 (59.1%) did not contain any floating plant species.

**Fig 1:**
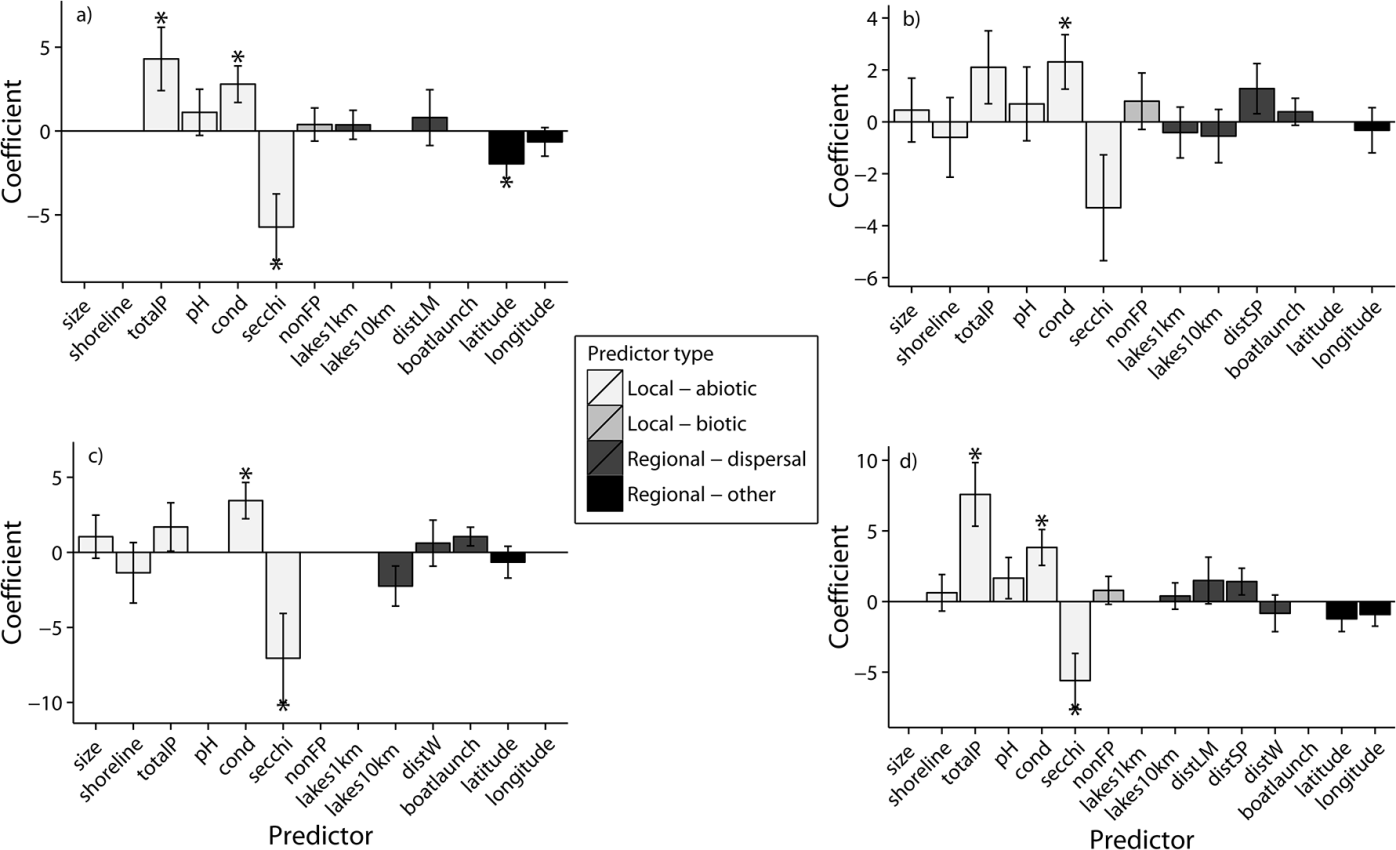
Coefficients of predictor variables from the model weighted averaged (ΔAIC_c_ < 2) generalized linear model. (a) *Lemna minor*, (b) *Spirodela polyrhiza*, (c) *Wolffia* sp., and (d) the floating plant functional group. Error bars are standard errors. Asterisk (*) indicates coefficients that were significantly greater than zero. Note: these coefficients are for variables that were scale from 0 to 1 prior to analysis. See Table 1 for variable codes.

The weighted average model for the occurrence of *L*. *minor* was composed of 6 models with ΔAIC_c_ < 2 and correctly predicted 69.7% of observations (optimal threshold for occurrence = 0.32; Kappa = 0.361, SD = 0.070, Table 3). Total phosphorus and conductivity had significant positive coefficients, while latitude and Secchi depth had significant negative coefficients (Fig 1A). The average model for the occurrence of *S*. *polyrhiza* was composed of 18 models and correctly predicted 68.0% of observations (optimal threshold for occurrence = 0.18; Kappa = 0.233, SD = 0.070, Table 3). Only conductivity had a significant positive coefficient (Fig 1B). The average model for *Wolffia* spp. was composed of 11 models and correctly predicted 75.4% of observations (optimal threshold for occurrence = 0.155; Kappa = 0.315, SD = 0.074, Table 3). Conductivity had a significant positive coefficient, while Secchi depth had a significant negative coefficient (Fig 1C). Finally, the average model for the presence of any floating plant species was composed of 27 models and correctly predicted 72.0% of observations (optimal threshold for occurrence = 0.39; Kappa = 0.431, SD = 0.068, Table 3). Conductivity and total phosphorus had significant positive coefficients and Secchi depth had a significant negative coefficient (Fig 1D).

**Table 3:** Confusion matrices for the model-averaged generalized linear models.

*Lemna minor*		**Observed**	
		**Absent**	**Present**
**Predicted**	**Absent**	83	36
	**Present**	17	39
*Spirodela polyrhiza*		**Observed**	
		**Absent**	**Present**
**Predicted**	**Absent**	99	45
	**Present**	11	20
*Wolffia* sp.		**Observed**	
		**Absent**	**Present**
**Predicted**	**Absent**	115	37
	**Present**	6	17
Floating plant presence		**Observed**	
		**Absent**	**Present**
**Predicted**	**Absent**	75	29
	**Present**	20	51

Model average of all models with Δ AIC_c_ < 2

**Fig 2:**
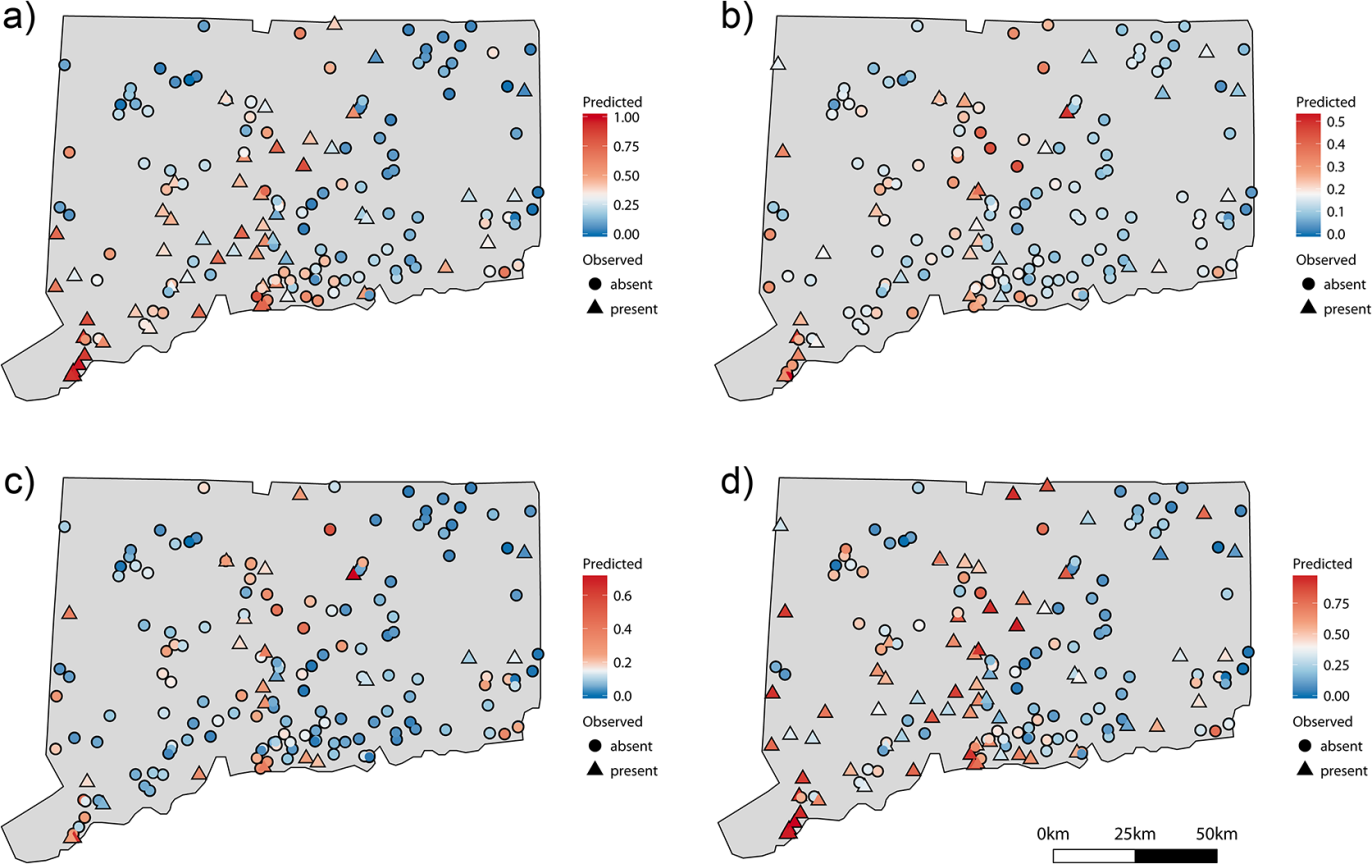
Observed and predicted floating plant occurrence from the model weighted averaged (ΔAIC_c_ < 2) generalized linear model. Observed presences (triangles) and absences (circles) and predicted presence and absence (red to blue). (a) *Lemna minor*, (b) *Spirodela polyrhiza*, (c) *Wolffia* sp., and (d) the floating plant functional group. The optimal threshold value to transform the probability of occurrence to a binary presence (1) or absence (0) is set to white on the color scale.

For all response variables, false negatives were more common than false positives (Fig 2). The total amount of explained variation (i.e., pseudo-R^2^ of the best model) ranged from 45.0% for floating plant presence to 13.7% for *S*. *polyrhiza* (35.7% for *L*. *minor* and 26.3% for *Wolffia* spp.). In general, models of floating plant species richness had similar results, with positive effects of total phosphorus and conductivity and negative effects of Secchi depth (pseudo-R^2^ 0.351, S1 File).

**Fig 3:**
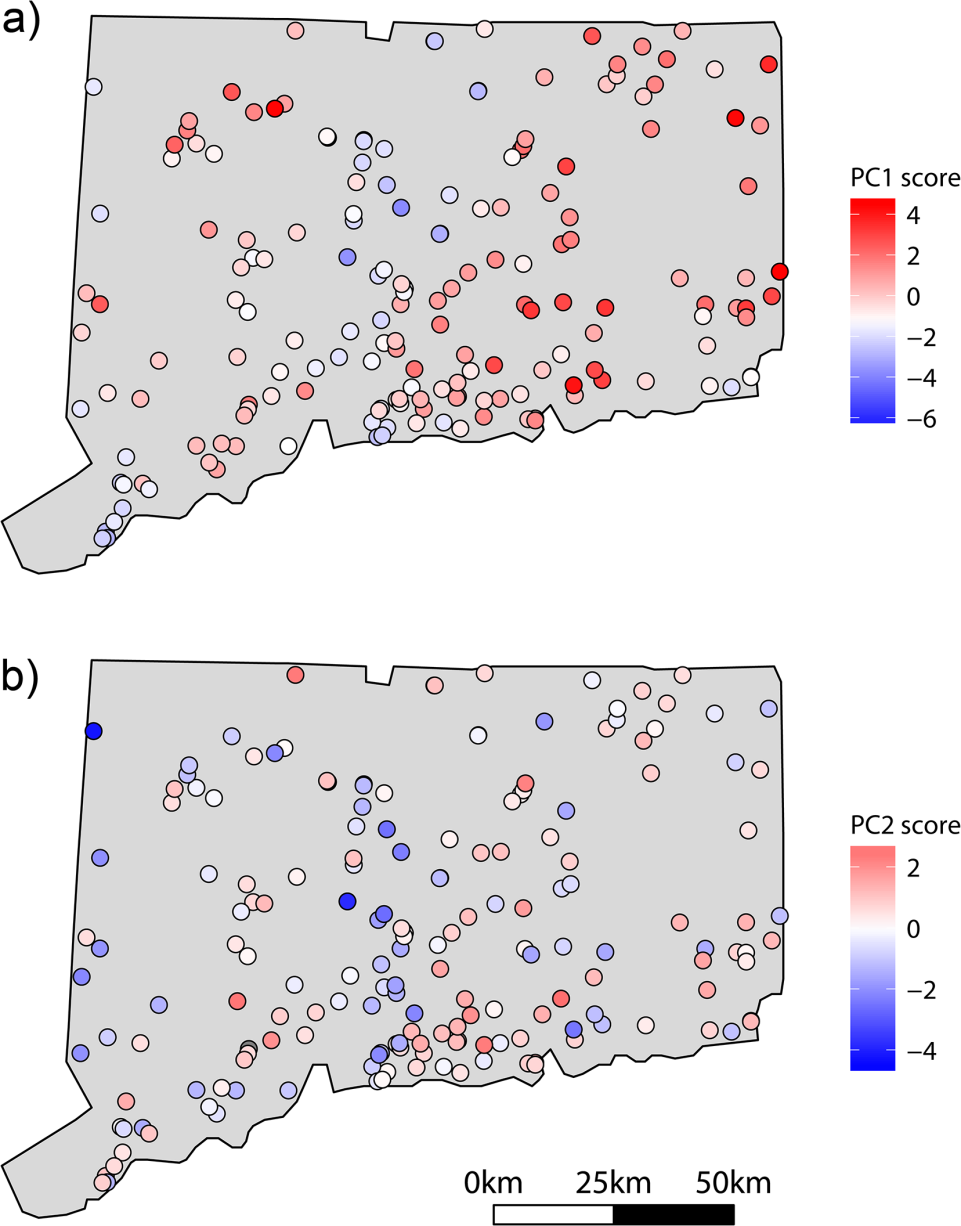
Scores for first two axes of the Principal Component Analysis of local water body conditions.

The first two principal components of local water body conditions explained approximately 53% of the variation in water body conditions (Table 4). Negative scores on PC1 were associated with alkalinity, pH, conductivity, and total phosphorus, while positive scores were associated with water body size, maximum depth, Secchi depth, and the richness of non-floating plant species. The largest values on PC2 were associated with total phosphorus while negative scores on PC2 were associated with water body size, maximum depth, pH, conductivity, and alkalinity (Table 4). Across Connecticut, negative scores on PC1 (i.e., high phosphorus, conductivity, pH, etc.) were generally found in the southern and central portions of the state, while positive PC1 scores (i.e., large, deep waterbodies) were found in the northwest and eastern portions of the state (Fig 3A). PC2 scores were distributed more sporadically throughout the state (Fig 3B).

**Table 4:** Axes of the Principal Component Analysis of environmental conditions in surveyed water bodies.

Variable	PC1	PC2	PC3	PC4
Water body surface area	0.353	-0.360	0.176	-0.179
Shoreline development index	0.239	-0.285	0.693	-0.084
Depth, maximum	0.373	-0.353	-0.374	-0.097
Richness of non-floating plants	0.409	-0.113	0.148	-0.058
Total phosphorus, average	-0.244	-0.055	-0.078	-0.916
pH, average	-0.238	-0.522	-0.007	0.329
Conductivity, average	-0.382	-0.361	-0.016	0.021
Alkalinity, average	-0.329	-0.472	-0.099	-0.004
Secchi depth	0.375	-0.154	-0.557	0.013
**Proportion of Variance**	0.346	0.186	0.117	0.101

### Permutation: Species pool size

**Fig 4:**
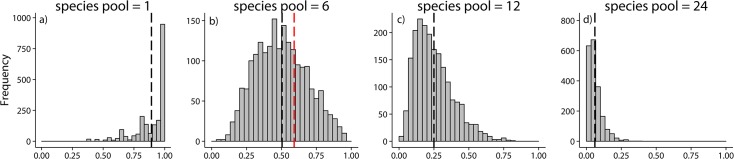
Expected proportion of water bodies without floating plants for a species pool various sizes. (a) one, (b) six, (c) 12, and (d) 24 species. Species occurrence was randomly drawn from the frequency distribution of the 124 aquatic plant taxa in Connecticut, USA (S2 Fig, S1 Table) and species were then randomly assigned to 176 water bodies. The proportion of water bodies without a floating plant species was calculated (n = 2000 iterations). Mean expected value is indicated by the dashed black line. Observed value in Connecticut is indicated by the dashed red line in (b) where the observed species pool is six.

The permutation test based on a random sampling of the occurrence of six floating plant species from the frequency of occurrence of all aquatic plant species (S2 Fig, S1 Table) found that the observed prevalence of the floating plant functional group was no different than expected by chance (Fig 4B). The expected distribution of water bodies without floating plants had a mean of 49.9% (95% confidence interval: 22.1–81.8%), while the observed percentage of water bodies without floating plants in Connecticut was 59.1%. A smaller species pool (i.e., one species) resulted in an expected 89.2% of water bodies without floating plants, while larger species pools of 12 or 24 species, resulted in an expectation of 25.0% and 6.0% water bodies without floating plants, respectively (Fig 4).

### Comparison to other regions

Of the eight other regions with floating plant prevalence data, richness of this functional group ranged from one species in Denmark to nine species in Finland (Table 2). For the five regions with sufficient data, Norway had the lowest percentage of water bodies without floating plants (20.3%), and Denmark had the highest percentage (81.7%) of waterbodies without floating plants (Table 2). Denmark was also the region that had the lowest species richness in this functional group.

## Discussion

In general, the presence of floating plants was best predicted by local, abiotic conditions. Water bodies with high nutrients and minerals (i.e., total phosphorus and conductivity) favored the occurrence of floating plants, whereas this functional group was less likely to occur in water bodies with clear water (i.e., high Secchi depth) and low nutrients. For most response variables, predictors related to dispersal (e.g., presence of a boat launch or distance to nearest occupied neighbor) were not important. *Lemna minor* was the only species with a significant spatial predictor (latitude), although the significant, negative effect of latitude on *L*. *minor* occurrence likely reflects a spatial pattern of abiotic conditions in Connecticut. Water bodies with conditions typically favoring the presence of floating plants (i.e., high conductivity, alkalinity, pH, total phosphorus or negative values on PC1 in Fig 3A) are mainly found along the southern coast of Connecticut or in the center of the state, along the Connecticut River. Northern water bodies, on either side of the Connecticut River, are typically less favorable for floating plants (i.e., large, deep water bodies with clear water or positive values on PC1 in Fig 3B). Geological factors (e.g., weathering of the drainage basin) or human population density (i.e., anthropogenic eutrophication) may contribute to this spatial pattern of abiotic conditions across the state. This result demonstrates how spatially patterned abiotic conditions can give the appearance that dispersal processes are contributing to species occurrence.

In other studies, similar abiotic conditions (i.e., alkalinity, nutrients, and other dissolved minerals) have been shown to be important predictors of floating plant occurrence [17, 36–37]. In addition to water chemistry parameters, water body properties such as the presence of an inlet or artificial pond enlargement can have a positive effect on floating plant richness [17], but were not considered in this study due to the nature of the database used. In an analysis of submerged and rooted-floating (i.e., lily) species in the same region as this study, Capers et al. [39] similarly found that the mineral content of the water (i.e., alkalinity) was positively correlated with species richness of aquatic plants, but also found that the intensity of human activity increased both native and invasive species richness, which was not evident for floating plants in this study. Unlike many other studies on the assembly of aquatic plant communities, this work focused on free-floating plants, a frequently-ignored group due to their small size. It also combined multiple lines of evidence (e.g., global comparisons and null model permutation tests) to gain a broader understanding.

Although community assembly depends on a combination of local and regional processes, local abiotic conditions frequently play a greater role in many aquatic systems including: macrophytes [14–15, 40–41], caddisflies [42], cladocerans and other zooplankton [41, 43], and fish [44], as was found in this study. Nevertheless, the relative strength of local environment and dispersal may depend on the spatial scale of the study [45] or may vary through time [44].

For all species and the floating plant functional group as a whole, a relatively large amount (approximately 55–86%) of variation was unexplained. The large amount of unexplained variation suggests that the presence of this functional group in a given lake or pond, is stochastic relative to local and regional predictors or alternatively that this analysis was missing important predictor variables that were not measured. This level of unexplained variation is common in studies of aquatic systems [14, 17, 39, 41]. Despite relatively low variance explained, the generalized linear models (GLM) had relatively low misclassification rates for observed data (approximately 25–30%, Table 3). Of the two types of misclassification errors, false-negatives (the model predicted that conditions were not suitable, but plants were present) were more common for all response variables. Assuming the model is correct; false-negatives could be attributed to non-equilibrium dynamics. Perhaps, the unsuitable conditions mean the plant is bound to go locally extinct, but has not done so yet. If false-positives (the model predicted presence, but absence was observed) were more common, then this could have indicated that dispersal limitation, where plants cannot get to otherwise suitable habitats, or random local extinctions were playing a role.

Although floating plants may be considered by some to be relatively uncommon as they occur in only approximately 40% of the lakes and ponds in Connecticut, the small size of the species pool of floating plants makes it likely that this functional group will be missing from many water bodies through random sampling alone (Fig 4). If this functional group had a greater number of species in this region, then we might expect it to be more common simply through random chance. Therefore, the biogeographic factors such as speciation and local extinction that shape the size of the floating plant species pool may influence the occurrence of this functional group in Connecticut ponds. Although the permutation used here presents a relatively simple model of community assembly, more complex alternatives can be extended from this approach. For example, rather than drawing floating plant occurrence (i.e., number of water bodies occupied) at random from the observed occurrence of all aquatic plant species, a weighted probability could be specified for species that rare or common relative to the average aquatic plant species. Also the process by which species are assigned waterbodies (“colonization”) could be modified to include a probability of species co-occurrence to incorporate models of community assembly based on competition (less-likely to co-occur) or niche similarity (more-likely to co-occur). Despite room for extending this permutation approach, the model presented here demonstrates how low species richness (i.e., a small species pool) can contribute to the rarity of a functional group.

The species richness and the pattern of occurrence of the floating plant functional group in Connecticut ponds are within the range observed in other regions of the world. In fact, in France, Denmark, and Washington, USA, the floating plant functional group is even less common than in the northeast USA. In all of these regions, the species richness of floating plants ranges from one to nine species, which is much smaller than the species pool of submerged plants (70 taxa) or emergent plants (40 taxa), but similar to the species pool of floating rooted plants (8 taxa), in Connecticut. A notable absence from this literature comparison is information on floating plant occurrence in tropical and sub-tropical lakes. In these warmer regions, floating plant species such as *Eichhornia crassipes* and *Salvinia molesta* can be especially nuisance species [46–47]. Floating plants can also play important ecological role in many South American lakes and ponds [48], but comparable data on the presence-absence of this group in these regions are not available.

Predicting the presence of the functional group as a whole, rather than individual species as is typical in other studies, explained more of the variance, but had similar prediction accuracy as the best individual species in this study. These results highlight the importance of evolutionary processes, which determine species pools, and how they interact with local conditions to determine the presence of a functional group within particular communities. Nevertheless, a large amount of variation remains unexplained when modeling the occurrence of floating plants. This study also demonstrates how spatially patterned abiotic conditions can give the erroneous appearance that dispersal processes are determining species occurrence.

## Supporting Information

S1 Dataset Data for presence/absence analysis. Includes summary statistics (min., mean, max., standard deviation, skewness, and kurtosis).
(CSV)

S1 Fig Correlation among predictor variables. Heat map of correlation matrix of predictor variables, consisting of Pearson product-moment correlations for all pairs, except for correlations with boat launch presence (“boatlaunch”), which were point-biserial correlations. Correlation matrix was fit with the hetcor function in the R package polycor.
(TIF)

S2 Fig Occurrence of aquatic plant species in 176 lakes and ponds in Connecticut, USA. Floating plant taxa are labelled. Taxonomic names can be found in S1 Table.
(TIF)

S1 File Results for GLMs of floating plant species richness. (PDF)

S1 Table Aquatic plant species in 176 lakes and ponds in Connecticut, USA. (DOCX)

## References

[1] Poff NL. Landscape filters and species traits: Towards mechanistic understanding and prediction in stream ecology. J. North Am. Benthol. Soc. 1997; 16: 391–409.

[2] Cottenie K. Integrating environmental and spatial processes in ecological community dynamics. Ecol. Lett. 2005; 8: 1175–1182. doi: 10.1111/j.1461-0248.2005.00820.x 21352441 10.1111/j.1461-0248.2005.00820.x

[3] Karst J, Gilbert B, Lechowicz MJ. Fern community assembly: The roles of chance and the environment at local and intermediate scales. Ecology 2005; 86: 2473–2486.

[4] Nhiwatiwa T, Brendonck L, Waterkeyn A, Vanschoenwinkel B. The importance of landscape and habitat properties in explaining instantaneous and long-term distributions of large branchiopods in subtropical temporary pans. Freshw. Biol. 2011; 56: 1992–2008.

[5] Mouquet N, Munguia P, Kneitel JM, Miller TE. Community assembly time and the relationship between local and regional species richness. Oikos 2003; 103: 618–626.

[6] Akasaka M, Takamura N. The relative importance of dispersal and the local environment for species richness in two aquatic plant growth forms. Oikos 2011; 120: 38–46.

[7] Beisner BE, Peres-Neto PR, Lindström ES, Barbettm A, Lorena Longhi M. The role of environmental and spatial processes in structuring lake communities from bacteria to fish. Ecology 2006; 87: 2985–2991. 17249222 10.1890/0012-9658(2006)87[2985:troeas]2.0.co;2

[8] Folke C, Carpenter S, Walker B, Scheffer M, Elmqvist T, Gunderson L, et al Regime shifts, resilience, and biodiversity in ecosystem management. Ann. Rev. Ecol. Evol. Syst. 2004; 35: 557–581.

[9] Vitousek PM, Walker LR, Whiteaker LD, Mueller-Dombois D, Matson PM. Biological invasion by *Myrica faya* alters ecosystem development in Hawaii. Science 1987; 238: 802–804. 17814707 10.1126/science.238.4828.802

[10] Elmqvist T, Folke C, Nyström M, Peterson G, Bengtsson J, Walker B, et al Response diversity, ecosystem change, and resilience. Fron. Ecol. Env. 2003; 1: 488–494.

[11] Stendera S, Adrian R, Bonada N, Cañedo-Argüelles M, Hugueny B, Januschke K, et al Drivers and stressors of freshwater biodiversity patterns across different ecosystems and scales: A review. Hydrobiologia 2012; 696: 1–28.

[12] Capers RS, Selsky R, Bugbee GJ. The relative importance of local conditions and regional processes in structuring aquatic plant communities. Freshw. Biol. 2010; 55: 952–966.

[13] Campbell RE, McIntosh AR. Do isolation and local habitat jointly limit the structure of stream invertebrate assemblages? Freshw. Biol. 2013; 58: 128–141.

[14] Mikulyuk A., Sharma S, Van Egeren S, Erdmann E, Nault ME, Hauxwell H. The relative role of environmental, spatial, and land-use patterns in explaining aquatic macrophyte community composition. Can. J. Fish. Aquat. Sci. 2011; 68: 1778–1789.

[15] Alahuhta J, Kanninen A, Hellsten S, Vuori K, Kuoppala M, Hämäläinen H. Variable response of functional macrophyte groups to lake characteristics, land use, and space: Implications for bioassessment. Hydrobiologia 2014; 737: 201–214.

[16] Li F, Chung N, Bae M, Kwon Y, Park Y. Relationships between stream macroinvertebrates and environmental variables at multiple spatial scales. Freshw. Biol. 2012; 57: 2107–2124.

[17] Edvardsen A, Økland RH. Variation in plant species richness in and adjacent to 64 ponds in SE Norweigan agricultural landscapes. Aquat. Bot. 2006; 85: 79–91.

[18] Lavorel S, Garnier E. Predicting changes in community composition and ecosystem functioning from plant traits: Revisiting the Holy Grail. Func. Ecol. 2002; 16: 545–556.

[19] Wetzel RG. Limnology: Lake and River Ecosystems. Elsevier; 2001.

[20] Dodds WK, Whiles MR. Freshwater Ecology: Concepts and Environmental Applications of Limnology. Elsevier; 2010.

[21] Morris K., Bailey PC, Boon PI, Hughes L. Alternative stable states in the aquatic vegetation of shallow urban lakes. II. Catastrophic loss of aquatic plants consequent to nutrient enrichment. Mar. Freshw. Res. 2003a; 54: 201–215.

[22] Morris K., Bailey PC, Boon PI, Hughes L. Alternative stable states in the aquatic vegetation of shallow urban lakes. I. Effects of plant harvesting and low-level nutrient enrichment. Mar. Freshw. Res. 2003b; 54: 185–200.

[23] Scheffer M, Szabó S, Gragnani A, van Nes EH, Rinaldi S, Kautsky N, et al Floating plant dominance as a stable state. Proc. Natl. Acad. Sci. USA 2003; 100: 4040–4045. 12634429 10.1073/pnas.0737918100 PMC153044

[24] Smith SDP. Identifying and evaluating causes of alternative community states in wetland plant communities. Oikos 2012; 121: 675–686.

[25] Janse JH, Van Puijenbroek PJTM. Effects of eutrophication in drainage ditches. Env. Poll. 1998; 102: 547–552.

[26] Jacobs DK. An ecological life-history of *Spirodela polyrhiza* (Greater duckweed) with emphasis on the turion phase. Ecol. Monogr. 1947; 17: 437–469.

[27] Keddy PA. Lakes as islands: The distributional ecology of two aquatic plants, *Lemna minor* L. and *L*. *trisulca* L. Ecology 1976; 57: 353–359.

[28] Burnham KP, Anderson DR. Model Selection and Multimodel Inference: A Practical Information-Theoretic Approach. Spring; 2002.

[29] Liu C, Berry PM, Dawson TP, Pearson RG. Selecting thresholds of occurrence in the prediction of species distributions. Ecography 2005; 28: 385–393.

[30] Liu C, White M, Newell G. Measuring and comparing the accuracy of species distribution models with presence-absence data. Ecography 2011; 34: 232–243.

[31] Nagelkerke NJD. A note on a general definition of the coefficient of determination. Biometrika 1991; 78: 691–692

[32] Cook RD. Influential observations in linear regression. J. Am. Stat. Assoc. 1979; 74: 169–174.

[33] Bornette G, Amoros C, Lamouroux N. Aquatic plant diversity in riverine wetlands: The role of connectivity. Freshw. Biol. 1998; 39: 267–283.

[34] Toivonen H, Huttunen P. Aquatic macrophytes and ecological gradients in 57 small lakes in southern Finland. Aquat. Bot. 1995; 51: 197–221.

[35] Trei T, Pall P. Macroflora in the watercourses of Saaremaa Island (Estonia). Boreal Environ. Res. 2004; 9: 25–35.

[36] Heegaard E, Birks HH, Gibson CE, Smith SJ, Wolfe-Murphy S. Species-environmental relationships of aquatic macrophytes in Northern Ireland. Aquat. Bot. 2001; 70: 175–223.

[37] Vestergaard O, Sand-Jensen K. Alkalinity and trophic state regulate aquatic plant distribution in Danish lakes. Aquat. Bot. 2000; 67: 85–107.

[38] Duigan C, Kovach W, Palmer M. Vegetation communities of British lakes: A revised classification. Joint Conservation Committee; 2006.

[39] Capers RS, Selsky R, Bugbee GJ, White JC. Species richness of both native and invasive aquatic plants influenced by environmental conditions and human activity. Botany 2009; 87: 306–314.

[40] Alahuhta J, Kanninen A, Hellsten S, Vuori K, Kuoppala M, Hämäläinen H. Environmental and spatial correlates of community composition, richness and status of boreal lake macrophytes. Ecol. Indic. 2013; 32: 172–181.

[41] Viana DS, Santamaría L, Schwenk K, Manca M, Hobæk A, Mjelde M, et al Environment and biogeography drive aquatic plant and cladoceran species richness across Europe. Freshw. Biol. 2014; 59: 2096–2106.

[42] Landeiro VL, Bini LM, Melo AS, Pes AMO, Mangnusson WE. The roles of dispersal limitation and environmental conditions in controlling caddisfly (Trichoptera) assemblages. Freshw. Biol. 2012; 57: 1554–1564.

[43] Cottenie K, Michels E, Nuytten N, De Meester L. Zooplankton metacommunity structure: Regional vs. local processes in highly interconnected ponds. Ecology 2003; 84: 991–1000.

[44] Erős T, Sály P, Takacs P, Specziár A, Bíró P. Temporal variability in the spatial and environmental determinants of functional metacommunity organization—stream fish in a human-modified landscape. Freshw. Biol. 2012; 57: 1914–1928.

[45] Rooney RC, Bayley SE. Relative influence of local- and landscape-level habitat quality on aquatic plant diversity in shallow open-water wetlands in Alberta’s boreal zone: Direct and indirect effects. Landscape Ecol. 2011; 26: 1023–1034.

[46] Schooler SS, Salau B, Julien MH, Ives AR. Alternative stable states explain unpredictable biological control of *Salvinia molesta* in Kakadu. Nature 2011; 470: 86–89. doi: 10.1038/nature09735 21293376 10.1038/nature09735

[47] Villamagna AM, Murphy BR. Ecological and socio-economic impacts of invasive water hyacinth (*Eichhornia crassipes*): A review. Freshw. Biol. 2010; 55: 282–298.

[48] De Tezanos Pinto P, O’Farrell I. Regime shifts between free-floating plants and phytoplankton: A review. Hydrobiologia 2014; 740: 13:24.

