# Supplementary figures and images for: Local and Regional Determinants of an Uncommon Functional Group in Freshwater Lakes and Ponds
Source: PLoS One. 2015 Jun 29;10(6):e0131980. doi: 10.1371/journal.pone.0131980 (PMC4488069; doi:10.1371/journal.pone.0131980)

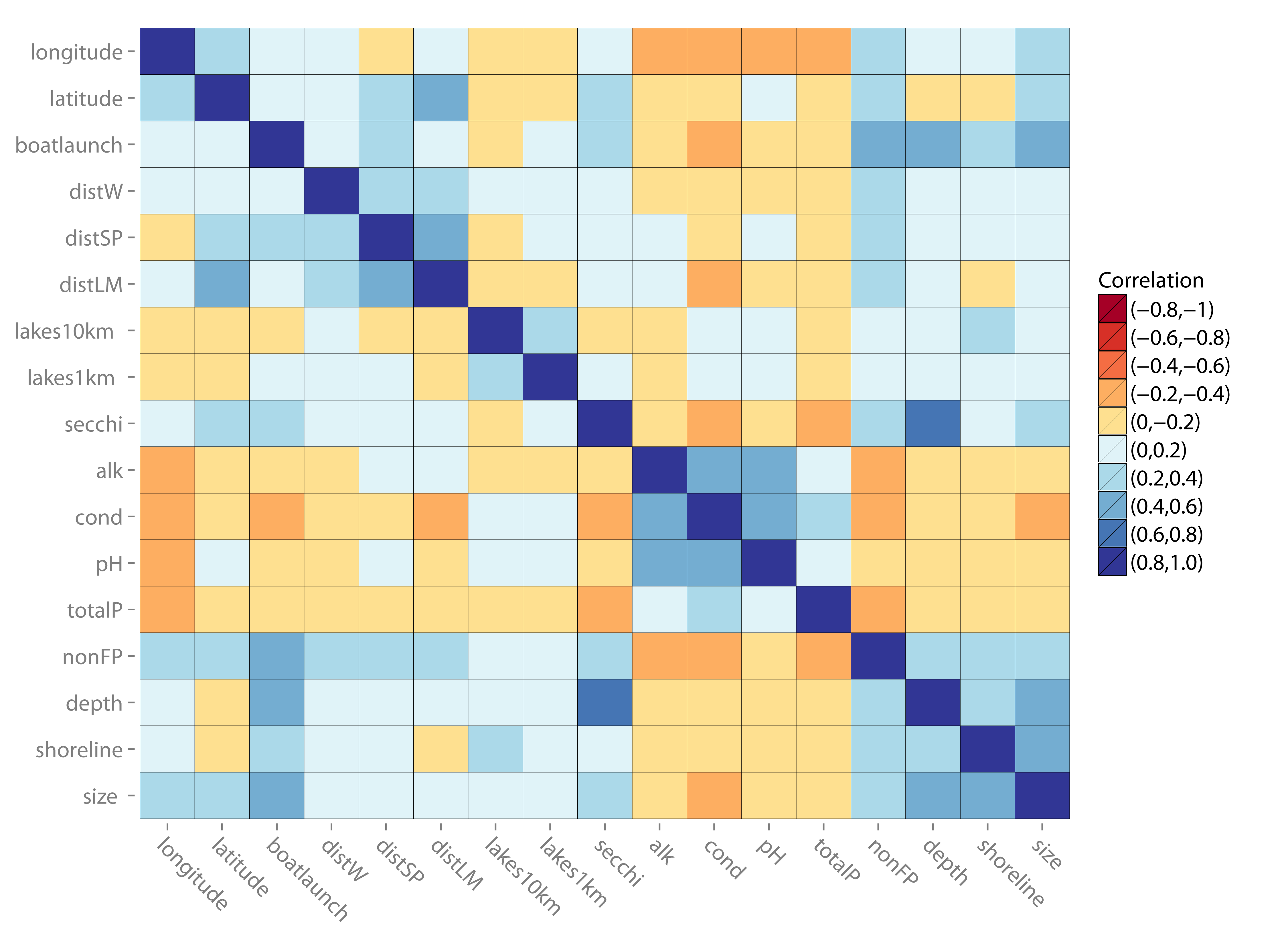

Supplement: S1 Fig — Heat map of correlation matrix of predictor variables, consisting of Pearson product-moment correlations for all pairs, except for correlations with boat launch presence (“boatlaunch”), which were point-biserial correlations. Correlation matrix was fit with the hetcor function in the R package polycor. (TIF) [file pone.0131980.s002.tif]

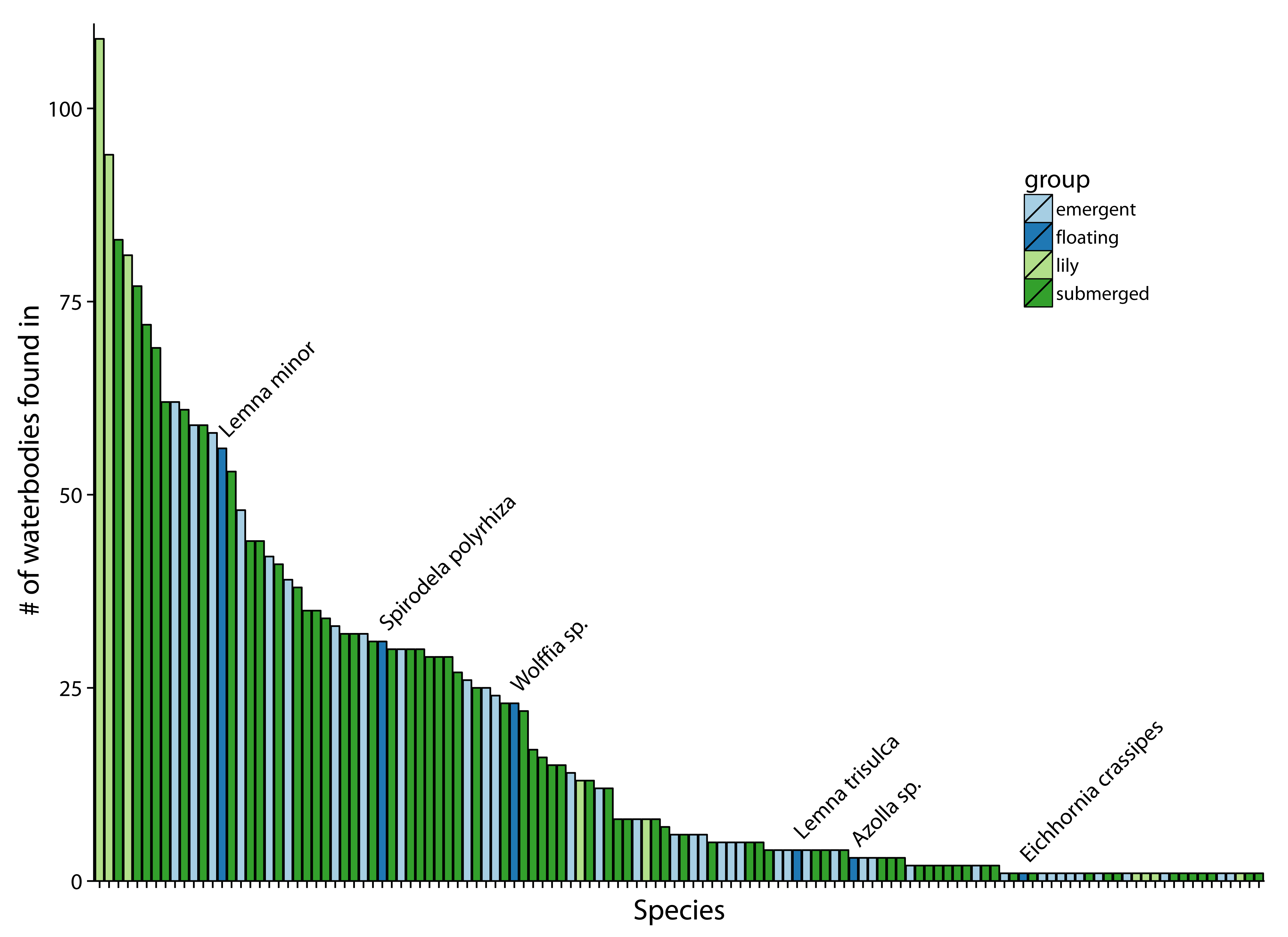

Supplement: S2 Fig — Floating plant taxa are labelled. Taxonomic names can be found in S1 Table. (TIF) [file pone.0131980.s003.tif]
