# Supplementary material for: Local and Regional Determinants of an Uncommon Functional Group in Freshwater Lakes and Ponds
Source: PLoS One. 2015 Jun 29;10(6):e0131980. doi: 10.1371/journal.pone.0131980 (PMC4488069; doi:10.1371/journal.pone.0131980)

S2 Appendix. Results of model averaging of generalized linear model for floating plant species richness.

The richness of floating plant species was modeled with a Poisson regression (Poisson error and log link functions). I used the distance to the nearest water body with *Lemna minor, Spirodela polyrhiza*, and *Wolffia* sp., respectively, as three separate predictor variables. All other details about model fitting and averaging are the same as in the main text.

Figure 1. Coefficients of predictor variable from the model weighted averaged (Δ $AIC_c < 2$) generalized linear model for floating plant species richness. Error bars are standard errors. Asterisk (*) indicates variables that are significant predictors. Note: these coefficients are for variables that were transformed prior to analysis. See Table 1 for variable codes.

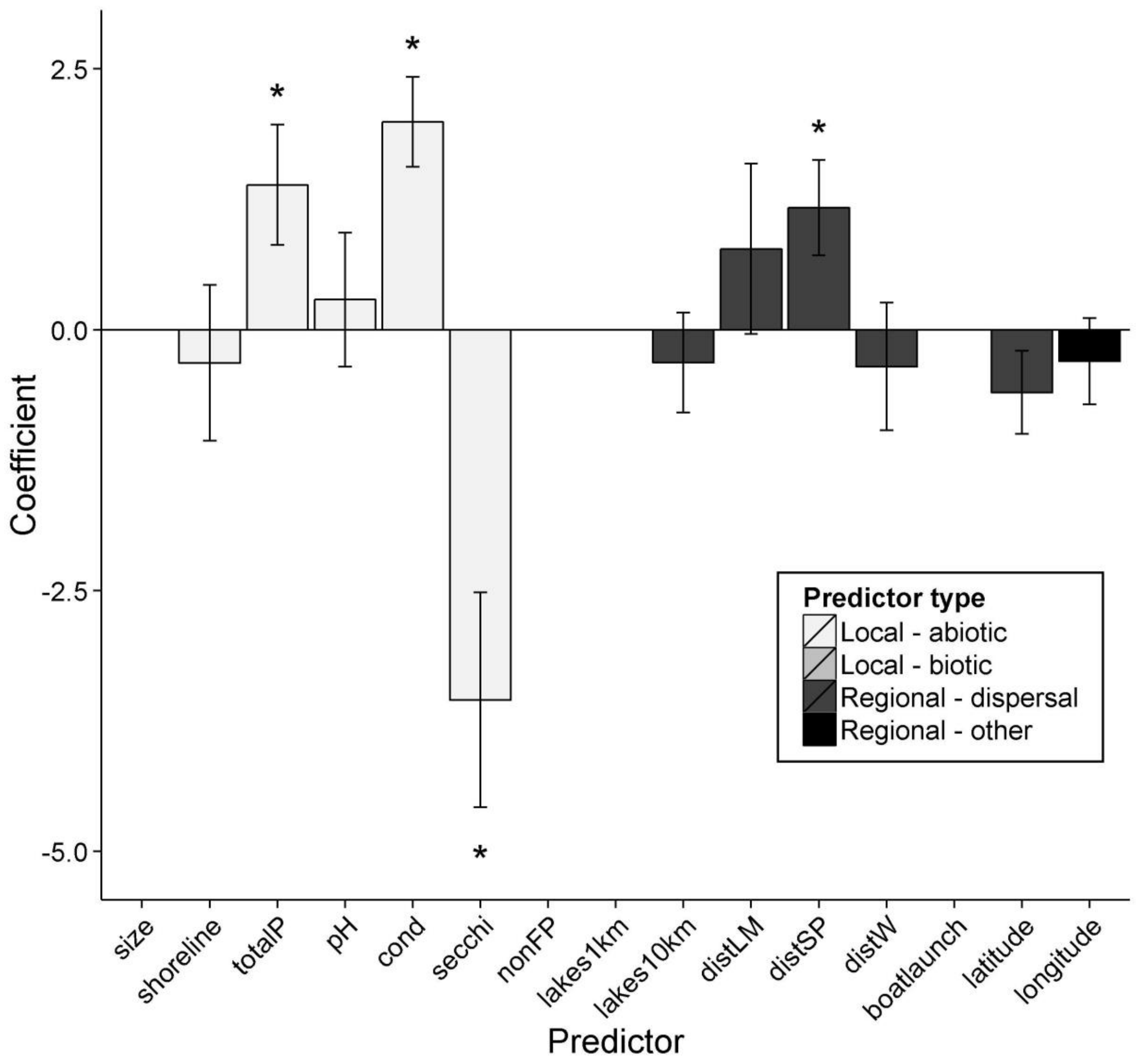

Figure 2. Map of observed (outer point) and predicted (inner point) floating plant species richness from the model weighted averaged (Δ $AIC_c < 2$) generalized linear model for floating plant species richness.

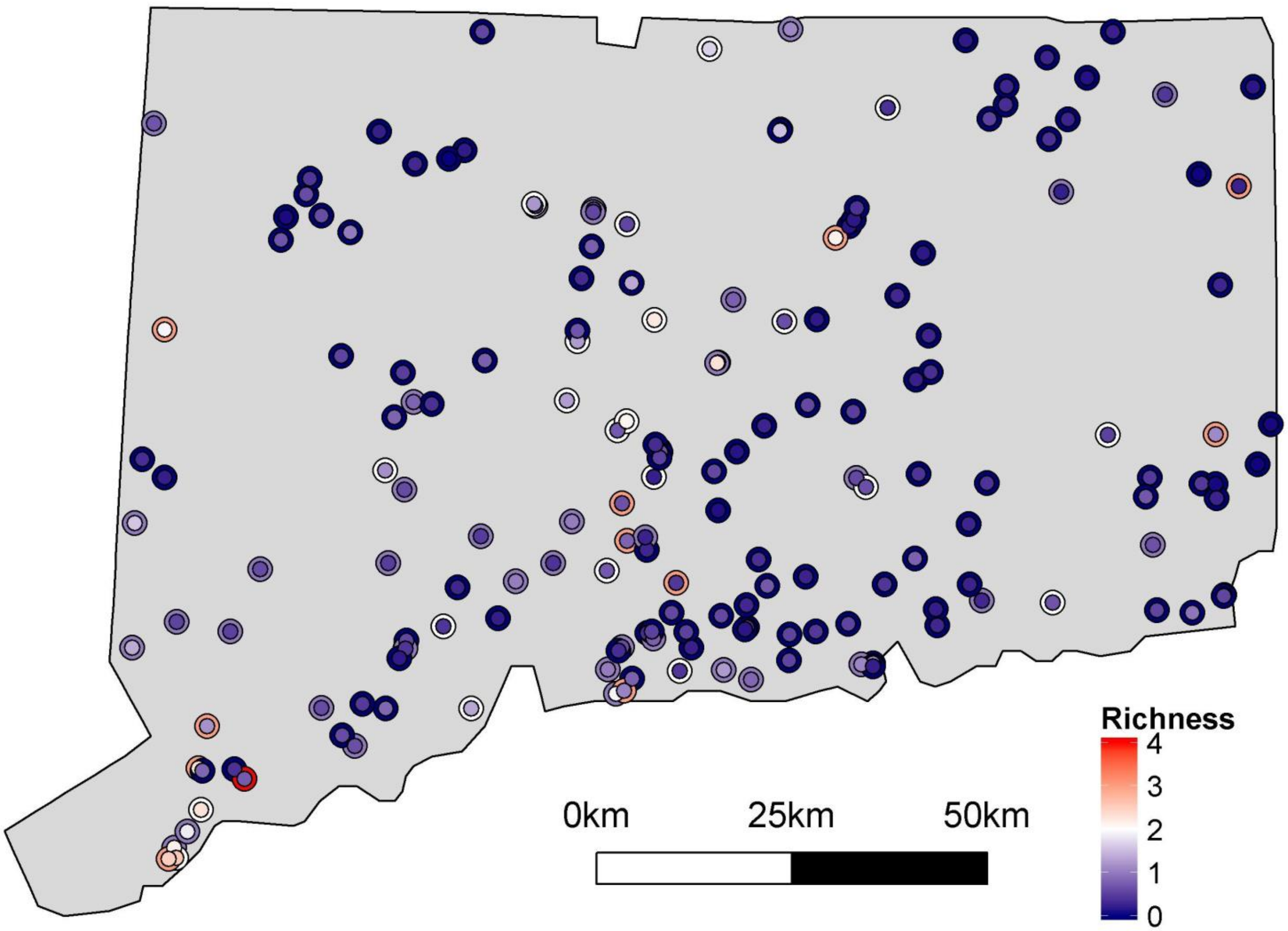

Supplement: S1 File — (PDF) [file pone.0131980.s004.pdf]
