# Supplementary material for: Local and Regional Determinants of an Uncommon Functional Group in Freshwater Lakes and Ponds
Source: PLoS One. 2015 Jun 29;10(6):e0131980. doi: 10.1371/journal.pone.0131980 (PMC4488069; doi:10.1371/journal.pone.0131980)
Supplement: S1 Table — (DOCX) [file pone.0131980.s005.docx]

**S4 Table. Aquatic plant species in 176 lakes and ponds in Connecticut, USA.**

| Taxa | Frequency | Functional group |
| --- | --- | --- |
| *Nymphaeae odorata* | 108 | Lily |
| *Nuphar variegata* | 93 | Lily |
| *Potamogeton biculpulatus* | 83 | Submerged |
| *Brasenia schreberi* | 80 | Lily |
| *Elodea nuttallii* | 77 | Submerged |
| *Potamogeton pusillus* | 71 | Submerged |
| *Najas flexilis* | 69 | Submerged |
| *Ceratophyllum demersum* | 62 | Submerged |
| *Pontederia cordata* | 62 | Emergent |
| *Utricularia gibba* | 61 | Submerged |
| *Eleocharis* sp. | 59 | Emergent |
| *Potamogeton epihydrus* | 59 | Submerged |
| *Sagittaria* sp. | 58 | Emergent |
| *Lemna minor* | 56 | Floating |
| *Utricularia purpurea* | 52 | Submerged |
| *Gratiola aurea* | 48 | Emergent |
| *Utricularia radiata* | 44 | Submerged |
| *Vallisneria americana* | 44 | Submerged |
| *Eriocaulon aquaticum* | 42 | Emergent |
| *Utricularia vulgaris* | 41 | Submerged |
| *Sparganium* sp. | 39 | Emergent |
| *Myriophyllum spicatum* | 38 | Submerged |
| *Elatine* sp. | 35 | Submerged |
| *Najas minor* | 35 | Submerged |
| *Potamogeton amplifolius* | 34 | Submerged |
| *Isoetes* sp. | 33 | Emergent |
| *Callitriche* sp. | 32 | Submerged |
| *Eleocharis acicularis* | 32 | Emergent |
| *Ceratophyllum echinatum* | 31 | Submerged |
| *Myriophyllum heterophyllum* | 31 | Submerged |
| *Spirodela polyrhiza* | 31 | Floating |
| *Elatine minima* | 30 | Submerged |
| *Ludwigia palustris* | 30 | Emergent |
| *Potamogeton foliosus* | 30 | Submerged |
| *Potamogeton pulcher* | 30 | Submerged |
| *Myriophyllum humile* | 29 | Submerged |
| *Potamogeton crispus* | 29 | Submerged |
| *Potamogeton natans* | 29 | Submerged |
| Charaphyte | 27 | Submerged |
| *Potamogeton robbinsii* | 25 | Submerged |
| Sedge | 25 | Emergent |
| *Typha* sp. | 25 | Emergent |
| *Ludwigia* sp. | 24 | Emergent |
| *Cabomba caroliniana* | 23 | Submerged |
| *Wolffia* sp. | 23 | Floating |
| *Najas gaudalupensis* | 22 | Submerged |
| *Elodea canadensis* | 16 | Submerged |
| *Utricularia macrorhiza* (FKA *vulgaris)* | 16 | Submerged |
| *Potamogeton gramineus* | 15 | Submerged |
| *Potamogeton perfoliatus* | 15 | Submerged |
| *Nymphoides cordata* | 13 | Lily |
| *Polygonum amphibium* | 13 | Emergent |
| *Potamogeton zosteriformis* | 13 | Submerged |
| *Alisma* sp. | 12 | Emergent |
| *Utricularia intermedia* | 12 | Submerged |
| *Glossostigma cleistanthum* | 8 | Submerged |
| *Myriophyllum tenellum* | 8 | Submerged |
| *Proserpinaca palustris* | 8 | Submerged |
| *Stuckenia pectinatus* | 8 | Submerged |
| *Utricularia minor* | 8 | Submerged |
| *Najas gracillima* | 7 | Submerged |
| *Decadon verticillatus* | 6 | Emergent |
| Filamentous algae | 6 | Submerged |
| *Peltandra virginica* | 6 | Emergent |
| *Ranunculus longirostris* | 6 | Emergent |
| *Callitriche heterophylla* | 5 | Submerged |
| *Juncus pelocarpus* | 5 | Emergent |
| *Lobelia dortmanna* | 5 | Emergent |
| *Phragmites australis* | 5 | Emergent |
| *Potamogeton praelongus* | 5 | Submerged |
| *Zosterella dubia* | 5 | Submerged |
| *Egeria densa* | 4 | Submerged |
| *Isoetes echinospora* | 4 | Emergent |
| *Juncus* sp. | 4 | Emergent |
| *Lemna trisulca* | 4 | Floating |
| *Polygonum* sp. | 4 | Emergent |
| *Potamogeton spirillus* | 4 | Submerged |
| *Sagittaria graminea* | 4 | Emergent |
| *Utricularia geminiscapa* | 4 | Submerged |
| *Azolla* sp. | 3 | Floating |
| *Isoetes engelmannii* | 3 | Emergent |
| *Isoetes tuckermannii* | 3 | Emergent |
| *Potamogeton ilinoensis* | 3 | Submerged |
| *Potamogeton oakesianus* | 3 | Submerged |
| *Potamogeton* sp. | 3 | Submerged |
| *Zannichellia palustris* | 3 | Submerged |
| *Cardamine* sp. | 2 | Emergent |
| *Hydrilla verticillata* | 2 | Submerged |
| *Myriophyllum aquaticum* | 2 | Submerged |
| *Myriophyllum heterophyllum* x *laxum* | 2 | Submerged |
| *Myriophyllum sibiricum* | 2 | Submerged |
| *Potamogeton palustris* | 2 | Submerged |
| *Potamogeton richardsonii* | 2 | Submerged |
| *Sagittaria latifolia* | 2 | Emergent |
| *Typha latifolia* | 2 | Emergent |
| *Utricularia* sp. | 2 | Submerged |
| *Alisma subcordatum* | 1 | Emergent |
| *Callitriche stagnalis* | 1 | Submerged |
| *Eicchornia crassipes* | 1 | Floating |
| *Elatine americana* | 1 | Submerged |
| *Eleocharis elliptica* | 1 | Emergent |
| *Isoetes acadiensis* | 1 | Emergent |
| *Isoetes lacustris* | 1 | Emergent |
| *Isoetes* x *eatonii* | 1 | Emergent |
| *Marsilea drummondii* | 1 | Emergent |
| *Megalondonta beckii* | 1 | Submerged |
| *Myosotis scorpiodes* | 1 | Emergent |
| *Myriophyllum alterniflorum* | 1 | Submerged |
| *Myriophyllum* sp. | 1 | Submerged |
| *Nasturtium* sp. | 1 | Emergent |
| *Nelumbo lutea* | 1 | Lily |
| *Nymphaeae odorata spp. tuberosa* | 1 | Lily |
| *Nymphoides* sp. | 1 | Lily |
| *Polygonum hydropiperoides* | 1 | Emergent |
| *Potamogeton berchtoldii* | 1 | Submerged |
| *Potamogeton confervoides* | 1 | Submerged |
| *Potamogeton ilinoensis* hybrid | 1 | Submerged |
| *Potamogeton nodosus* | 1 | Submerged |
| *Potamogeton obtusifolius* | 1 | Submerged |
| *Schoenoplectus tabernaemontani* | 1 | Emergent |
| *Sparganium americanum* | 1 | Emergent |
| *Trapa natans* | 1 | Lily |
| *Utricularia minima* | 1 | Submerged |
| *Utricularia subulata* | 1 | Submerged |
